# 4-(2,3-Dihydroxybenzyl­ideneamino)-3-methyl-1*H*-1,2,4-triazol-5(4*H*)-one

**DOI:** 10.1107/S1600536809045772

**Published:** 2009-11-07

**Authors:** Hasan Tanak, Yavuz Köysal, Metin Yavuz, Şamil Işık, Gülşah Gül

**Affiliations:** a Department of Physics, Ondokuz Mayıs University, TR-55139 Samsun, Turkey; b Samsun Vocational School, Ondokuz Mayıs University, TR-55139 Samsun, Turkey; c Department of Physics, Faculty of Arts & Science, Ondokuz Mayıs University, TR-55139 Kurupelit-Samsun, Turkey; d Department of Physics, Ondokuz Mayıs University, TR-55139 Samsun, Turkey; e Department of Chemistry, Karadeniz Tecnical University, Trabzon, Turkey

## Abstract

All the non-H atoms of the title compound, C_10_H_10_N_4_O_3_, are almost coplanar, the maximum deviation from planarity being 0.065 (3) Å. The dihedral angle between the aromatic rings is 1.66 (6)°. The mol­ecule adopts the enol–imine tautomeric form with an intra­molecular hydrogen-bonding inter­action between the Schiff base N atom and the hydr­oxy group. In the crystal, inter­molecular N—H⋯O and O—H⋯O hydrogen bonds link the mol­ecules into a three-dimensional network.

## Related literature

For the synthesis of the title compound, see Ünver *et al.* (2008 ▶). For related compounds, see: Köysal *et al.* (2007 ▶); Tanak *et al.*, (2009 ▶). For hydrogen-bond motifs, see Bernstein *et al.* (1995 ▶).
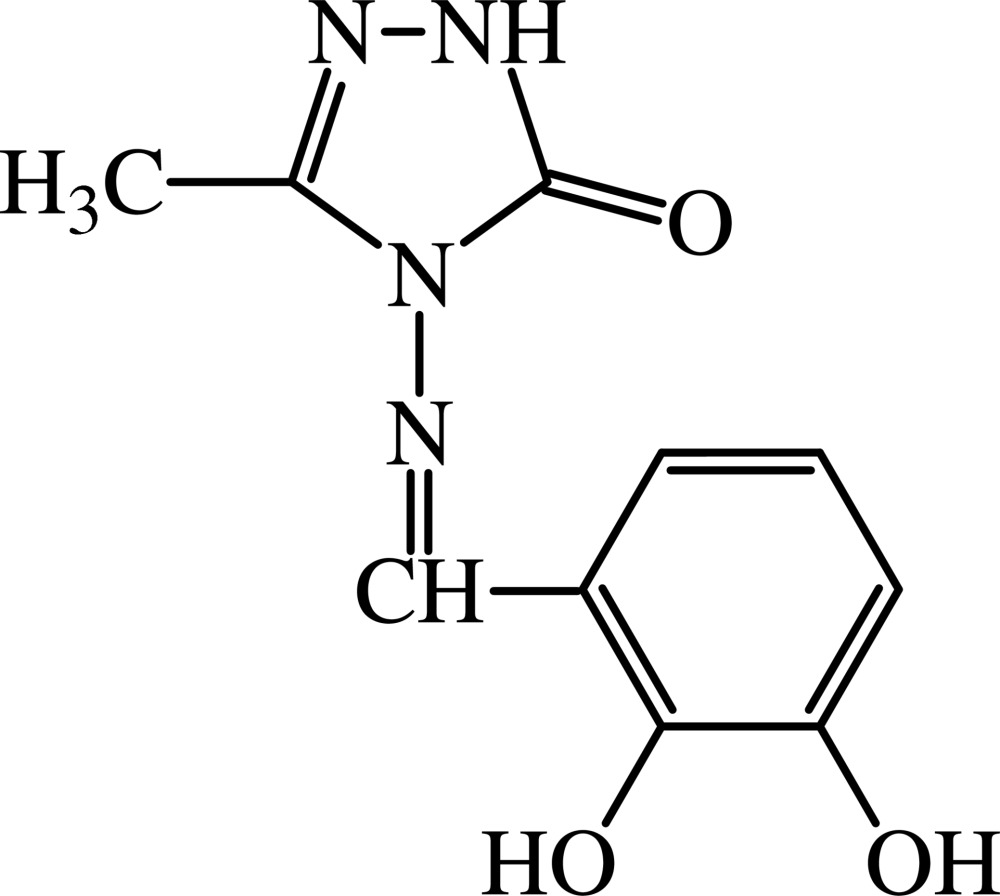


## Experimental

### 

#### Crystal data

C_10_H_10_N_4_O_3_
*M* *_r_* = 234.22
Monoclinic, 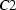

*a* = 13.944 (3) Å
*b* = 6.2551 (7) Å
*c* = 11.882 (2) Å
β = 93.857 (17)°
*V* = 1034.0 (3) Å^3^
*Z* = 4
Mo *K*α radiation
μ = 0.12 mm^−1^
*T* = 296 K
0.60 × 0.42 × 0.20 mm

#### Data collection

Stoe IPDS II diffractometer
Absorption correction: integration (*X-RED32*; Stoe & Cie, 2002 ▶) *T*_min_ = 0.944, *T*_max_ = 0.974
3000 measured reflections
1118 independent reflections
1058 reflections with *I* > 2σ(*I*)
*R*_int_ = 0.036

#### Refinement

*R*[*F* ^2^ > 2σ(*F* ^2^)] = 0.038
*wR*(*F* ^2^) = 0.116
*S* = 1.09
1118 reflections
159 parameters
1 restraint
H atoms treated by a mixture of independent and constrained refinement
Δρ_max_ = 0.31 e Å^−3^
Δρ_min_ = −0.27 e Å^−3^

### 

Data collection: *X-AREA* (Stoe & Cie, 2002 ▶); cell refinement: *X-AREA*; data reduction: *X-RED32* (Stoe & Cie, 2002 ▶); program(s) used to solve structure: *SHELXS97* (Sheldrick, 2008 ▶); program(s) used to refine structure: *SHELXL97* (Sheldrick, 2008 ▶); molecular graphics: *ORTEP-3 for Windows* (Farrugia, 1997 ▶); software used to prepare material for publication: *WinGX* (Farrugia, 1999 ▶).

## Supplementary Material

Crystal structure: contains datablocks I, global. DOI: 10.1107/S1600536809045772/bt5118sup1.cif

Structure factors: contains datablocks I. DOI: 10.1107/S1600536809045772/bt5118Isup2.hkl

Additional supplementary materials:  crystallographic information; 3D view; checkCIF report

## Figures and Tables

**Table 1:** Hydrogen-bond geometry (Å, °)

*D*—H⋯*A*	*D*—H	H⋯*A*	*D*⋯*A*	*D*—H⋯*A*
O2—H2⋯O1^i^	0.82	2.11	2.842 (3)	148
N3—H3⋯O3^ii^	0.86	2.00	2.830 (3)	163
O1—H1⋯N1	0.87 (5)	1.85 (5)	2.634 (3)	148 (4)

Symmetry codes: (i) 
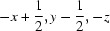
; (ii) 
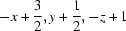
.

**Fig. 1.**
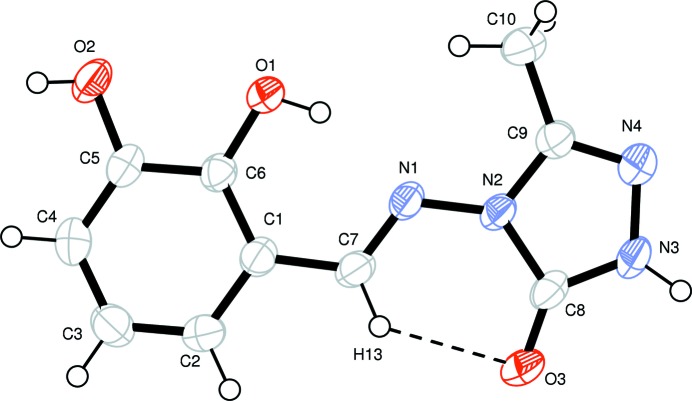
A view of the title compound, with the atom-numbering scheme and 50% probability displacement ellipsoids. The dashed line indicates the intramolecular hydrogen bond.

**Fig. 2.**
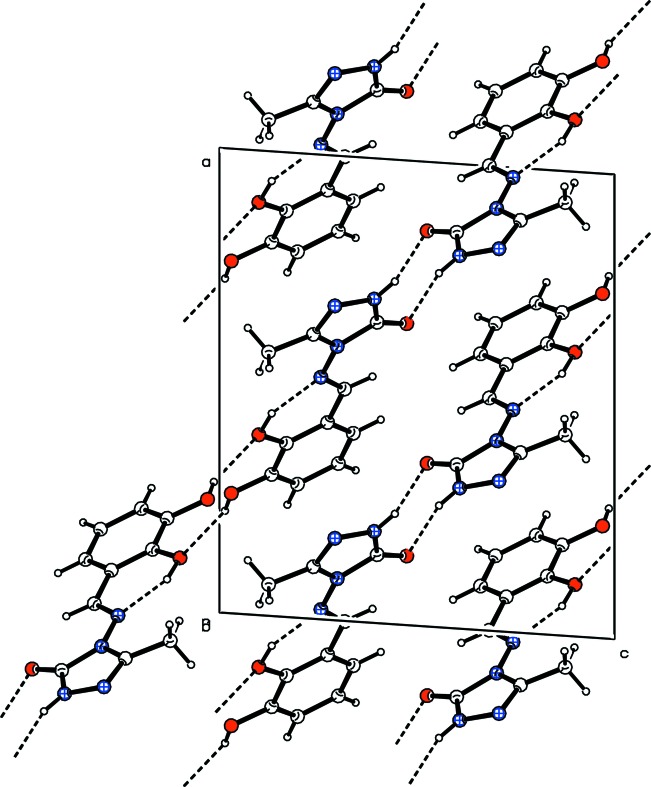
A partial packing of the title compound.

## supplementary crystallographic information

### Comment

The 1,2,4-triazole ring is strictly planar and the dihedral angle between the aromatic ring systems [C1/C6 and C8/N2] is 1.66 (6)°. The phenol H atom forms a strong intramolecular hydrogen bond with the imine N atom which is consistent with related structures (Köysal *et al.*, 2007; Tanak *et al.*, 2009), Fig 2.

The torsion angle C1—C7—N1—N2, bridged the aromatic ring systems, is 178.5 (2)° shows that for the title compound, the side chain conformation induced by the anti conformations, respectively. The interatomic distances within the triazole ring of are not equal. The C9—N4 is double bond and shorter than the conjugated C8—N2 and C9—N2 bonds. The length of the C7=N1 double bond is 1.278 (3) Å, it is almost consistent with standars 1.28 value of C=N double bond. The imino group is coplanar with the hydroxyphenyl ring as it can be shown by the C6—C1—C7—N1 torsion angle is -1.6 (3) Å.

Compound (I) is stabilized by N—H···O and O—H···O intermolecular contacts which link the molecules infinite chain and O—H···O, C—H···O and C—H···N type intramolecular hydrogen bonds. N3—H3···O3 (symmetry code: -*x* + 3/2,*y* + 1/2,-*z* + 1) bond is generates eight-membered ring, producing a *R*_2_^2^(8) motif (Bernstein, *et al.*, 1995). An intramolecular O1—H1···N1 hydrogen bond generates a six-membered ring, producing a S(6) ring motif (Bernstein, *et al.*, 1995), resulting in approximate planarity of the molecular skeleton [O···N= 2.636 (2) Å].

### Experimental

The title compound, C_10_H_10_N_4_O_3_, was synthesized according to the method of Ünver *et al.* (2008).

### Refinement

H atoms were refined using a riding model with C_aromatic_—H = 0.93 Å, C_methyl_—H = 0.96 Å. 830 Friedel-related reflections were merged in the final refinement because of the meaningless value of the absolute structure parameter (Flack, 1983).

### Crystal data

**Table:**

C_10_H_10_N_4_O_3_	*F*(000) = 488
*M**_r_* = 234.22	*D*_x_ = 1.505 Mg m^−^^3^
Monoclinic, *C*2	Mo *K*α radiation, λ = 0.71073 Å
Hall symbol: C 2y	Cell parameters from 4561 reflections
*a* = 13.944 (3) Å	θ = 1.7–28.0°
*b* = 6.2551 (7) Å	µ = 0.12 mm^−^^1^
*c* = 11.882 (2) Å	*T* = 296 K
β = 93.857 (17)°	Prism., yellow
*V* = 1034.0 (3) Å^3^	0.60 × 0.42 × 0.20 mm
*Z* = 4	

### Data collection

**Table:**

Stoe IPDS II diffractometer	1118 independent reflections
Radiation source: fine-focus sealed tube	1058 reflections with *I* > 2σ(*I*)
graphite	*R*_int_ = 0.036
Detector resolution: 6.67 pixels mm^-1^	θ_max_ = 26.0°, θ_min_ = 1.7°
rotation method scans	*h* = −17→17
Absorption correction: integration (*X-RED32*; Stoe & Cie, 2002)	*k* = −7→7
*T*_min_ = 0.944, *T*_max_ = 0.974	*l* = −14→12
3000 measured reflections	

### Refinement

**Table:**

Refinement on *F*^2^	Secondary atom site location: difference Fourier map
Least-squares matrix: full	Hydrogen site location: inferred from neighbouring sites
*R*[*F*^2^ > 2σ(*F*^2^)] = 0.038	H atoms treated by a mixture of independent and constrained refinement
*wR*(*F*^2^) = 0.116	*w* = 1/[σ^2^(*F*_o_^2^) + (0.0817*P*)^2^ + 0.2531*P*] where *P* = (*F*_o_^2^ + 2*F*_c_^2^)/3
*S* = 1.09	(Δ/σ)_max_ < 0.001
1118 reflections	Δρ_max_ = 0.31 e Å^−^^3^
159 parameters	Δρ_min_ = −0.27 e Å^−^^3^
1 restraint	Extinction correction: *SHELXL97* (Sheldrick, 2008), Fc^*^=kFc[1+0.001xFc^2^λ^3^/sin(2θ)]^-1/4^
Primary atom site location: structure-invariant direct methods	Extinction coefficient: 0.014 (3)

### Special details

**Table:**

Geometry. All e.s.d.'s (except the e.s.d. in the dihedral angle between two l.s. planes) are estimated using the full covariance matrix. The cell e.s.d.'s are taken into account individually in the estimation of e.s.d.'s in distances, angles and torsion angles; correlations between e.s.d.'s in cell parameters are only used when they are defined by crystal symmetry. An approximate (isotropic) treatment of cell e.s.d.'s is used for estimating e.s.d.'s involving l.s. planes.
Refinement. Refinement of *F*^2^ against ALL reflections. The weighted *R*-factor *wR* and goodness of fit *S* are based on *F*^2^, conventional *R*-factors *R* are based on *F*, with *F* set to zero for negative *F*^2^. The threshold expression of *F*^2^ > σ(*F*^2^) is used only for calculating *R*-factors(gt) *etc*. and is not relevant to the choice of reflections for refinement. *R*-factors based on *F*^2^ are statistically about twice as large as those based on *F*, and *R*- factors based on ALL data will be even larger.

### Fractional atomic coordinates and isotropic or equivalent isotropic displacement parameters (Å^2^)

**Table:**

	*x*	*y*	*z*	*U*_iso_*/*U*_eq_	
C1	0.42468 (17)	0.3934 (5)	0.2738 (2)	0.0359 (6)	
C2	0.4051 (2)	0.2125 (6)	0.3388 (2)	0.0437 (7)	
H8	0.4384	0.1927	0.4084	0.052*	
C3	0.3379 (2)	0.0655 (5)	0.3012 (3)	0.0468 (7)	
H9	0.3259	−0.0535	0.3451	0.056*	
C4	0.28754 (19)	0.0937 (5)	0.1977 (3)	0.0423 (7)	
H44	0.2419	−0.0067	0.1722	0.051*	
C5	0.30481 (17)	0.2698 (5)	0.1325 (2)	0.0367 (6)	
C6	0.37377 (17)	0.4209 (5)	0.1696 (2)	0.0339 (6)	
C7	0.49894 (18)	0.5409 (5)	0.3167 (2)	0.0375 (6)	
H13	0.5301	0.5190	0.3874	0.045*	
C8	0.64752 (17)	0.8427 (5)	0.3996 (2)	0.0369 (6)	
C9	0.61215 (17)	1.0237 (5)	0.2381 (2)	0.0367 (6)	
C10	0.5679 (2)	1.0718 (6)	0.1242 (2)	0.0463 (7)	
H15A	0.5240	0.9595	0.1009	0.069*	
H15B	0.6172	1.0821	0.0718	0.069*	
H15C	0.5338	1.2049	0.1259	0.069*	
N1	0.52117 (14)	0.7007 (4)	0.25680 (18)	0.0346 (5)	
N2	0.58991 (14)	0.8440 (4)	0.29821 (17)	0.0347 (5)	
N3	0.69709 (17)	1.0251 (5)	0.3919 (2)	0.0441 (6)	
H3	0.7380	1.0693	0.4442	0.053*	
N4	0.67653 (16)	1.1360 (5)	0.2924 (2)	0.0436 (6)	
O1	0.38826 (15)	0.5879 (4)	0.09998 (18)	0.0464 (6)	
O2	0.25821 (14)	0.3108 (4)	0.03004 (16)	0.0478 (6)	
H2	0.2197	0.2146	0.0141	0.072*	
O3	0.64859 (14)	0.7078 (4)	0.47417 (16)	0.0471 (5)	
H1	0.438 (3)	0.656 (9)	0.131 (4)	0.074 (13)*	

### Atomic displacement parameters (Å^2^)

**Table:**

	*U*^11^	*U*^22^	*U*^33^	*U*^12^	*U*^13^	*U*^23^
C1	0.0356 (12)	0.0356 (15)	0.0357 (12)	−0.0006 (11)	−0.0031 (10)	−0.0023 (12)
C2	0.0501 (15)	0.0436 (17)	0.0362 (13)	−0.0019 (14)	−0.0062 (11)	0.0060 (14)
C3	0.0557 (16)	0.0408 (17)	0.0446 (15)	−0.0055 (14)	0.0084 (12)	0.0037 (13)
C4	0.0385 (13)	0.0375 (15)	0.0507 (16)	−0.0066 (12)	0.0007 (11)	−0.0069 (13)
C5	0.0301 (11)	0.0395 (16)	0.0396 (13)	0.0018 (11)	−0.0040 (10)	−0.0078 (11)
C6	0.0325 (11)	0.0311 (13)	0.0371 (12)	0.0012 (11)	−0.0041 (9)	−0.0008 (11)
C7	0.0393 (12)	0.0398 (16)	0.0322 (11)	0.0013 (12)	−0.0072 (9)	−0.0004 (12)
C8	0.0327 (11)	0.0408 (14)	0.0354 (12)	0.0039 (11)	−0.0101 (9)	−0.0046 (12)
C9	0.0360 (12)	0.0369 (14)	0.0365 (12)	0.0004 (11)	−0.0031 (9)	0.0007 (12)
C10	0.0431 (14)	0.0524 (19)	0.0420 (14)	−0.0001 (13)	−0.0081 (11)	0.0084 (14)
N1	0.0334 (10)	0.0329 (12)	0.0360 (10)	−0.0011 (9)	−0.0096 (8)	−0.0030 (10)
N2	0.0326 (10)	0.0359 (12)	0.0339 (10)	−0.0007 (9)	−0.0107 (8)	−0.0002 (10)
N3	0.0433 (11)	0.0432 (14)	0.0431 (12)	−0.0087 (11)	−0.0172 (9)	−0.0034 (12)
N4	0.0402 (11)	0.0448 (14)	0.0445 (12)	−0.0061 (11)	−0.0077 (9)	0.0013 (12)
O1	0.0493 (11)	0.0393 (12)	0.0472 (11)	−0.0091 (10)	−0.0206 (9)	0.0101 (9)
O2	0.0463 (10)	0.0471 (13)	0.0470 (12)	−0.0054 (10)	−0.0183 (9)	−0.0041 (10)
O3	0.0477 (10)	0.0514 (13)	0.0392 (10)	−0.0007 (10)	−0.0178 (8)	0.0063 (10)

### Geometric parameters (Å, °)

**Table:**

C1—C6	1.396 (4)	C8—O3	1.223 (4)
C1—C2	1.407 (4)	C8—N3	1.340 (4)
C1—C7	1.453 (4)	C8—N2	1.402 (3)
C2—C3	1.367 (4)	C9—N4	1.280 (4)
C2—H8	0.9300	C9—N2	1.378 (4)
C3—C4	1.386 (4)	C9—C10	1.480 (4)
C3—H9	0.9300	C10—H15A	0.9600
C4—C5	1.377 (4)	C10—H15B	0.9600
C4—H44	0.9300	C10—H15C	0.9600
C5—O2	1.365 (3)	N1—N2	1.379 (3)
C5—C6	1.398 (4)	N3—N4	1.384 (4)
C6—O1	1.356 (3)	N3—H3	0.8600
C7—N1	1.277 (4)	O1—H1	0.87 (5)
C7—H13	0.9300	O2—H2	0.8200
			
C6—C1—C2	118.7 (2)	O3—C8—N2	127.2 (3)
C6—C1—C7	122.7 (2)	N3—C8—N2	101.8 (2)
C2—C1—C7	118.6 (2)	N4—C9—N2	111.1 (2)
C3—C2—C1	121.1 (3)	N4—C9—C10	125.8 (3)
C3—C2—H8	119.5	N2—C9—C10	123.0 (3)
C1—C2—H8	119.5	C9—C10—H15A	109.5
C2—C3—C4	120.0 (3)	C9—C10—H15B	109.5
C2—C3—H9	120.0	H15A—C10—H15B	109.5
C4—C3—H9	120.0	C9—C10—H15C	109.5
C5—C4—C3	120.3 (3)	H15A—C10—H15C	109.5
C5—C4—H44	119.9	H15B—C10—H15C	109.5
C3—C4—H44	119.9	C7—N1—N2	119.9 (2)
O2—C5—C4	124.1 (2)	N1—N2—C9	121.4 (2)
O2—C5—C6	115.5 (3)	N1—N2—C8	130.3 (2)
C4—C5—C6	120.4 (2)	C9—N2—C8	108.3 (2)
O1—C6—C1	123.3 (2)	C8—N3—N4	114.0 (2)
O1—C6—C5	117.1 (2)	C8—N3—H3	123.0
C1—C6—C5	119.6 (3)	N4—N3—H3	123.0
N1—C7—C1	119.7 (2)	C9—N4—N3	104.7 (2)
N1—C7—H13	120.2	C6—O1—H1	105 (3)
C1—C7—H13	120.2	C5—O2—H2	109.5
O3—C8—N3	131.0 (2)		
			
C6—C1—C2—C3	0.2 (4)	C1—C7—N1—N2	178.4 (2)
C7—C1—C2—C3	−178.2 (3)	C7—N1—N2—C9	−176.8 (2)
C1—C2—C3—C4	−0.2 (5)	C7—N1—N2—C8	3.3 (4)
C2—C3—C4—C5	−0.1 (4)	N4—C9—N2—N1	179.0 (2)
C3—C4—C5—O2	−179.6 (3)	C10—C9—N2—N1	−2.6 (4)
C3—C4—C5—C6	0.5 (4)	N4—C9—N2—C8	−1.1 (3)
C2—C1—C6—O1	−179.0 (2)	C10—C9—N2—C8	177.3 (3)
C7—C1—C6—O1	−0.7 (4)	O3—C8—N2—N1	0.5 (5)
C2—C1—C6—C5	0.2 (4)	N3—C8—N2—N1	−178.5 (3)
C7—C1—C6—C5	178.5 (2)	O3—C8—N2—C9	−179.4 (3)
O2—C5—C6—O1	−1.2 (4)	N3—C8—N2—C9	1.6 (3)
C4—C5—C6—O1	178.7 (2)	O3—C8—N3—N4	179.4 (3)
O2—C5—C6—C1	179.5 (2)	N2—C8—N3—N4	−1.7 (3)
C4—C5—C6—C1	−0.5 (4)	N2—C9—N4—N3	0.0 (3)
C6—C1—C7—N1	−1.6 (4)	C10—C9—N4—N3	−178.3 (3)
C2—C1—C7—N1	176.7 (3)	C8—N3—N4—C9	1.1 (3)

### Hydrogen-bond geometry (Å, °)

**Table:**

*D*—H···*A*	*D*—H	H···*A*	*D*···*A*	*D*—H···*A*
O2—H2···O1^i^	0.82	2.11	2.842 (3)	148
N3—H3···O3^ii^	0.86	2.00	2.830 (3)	163
O1—H1···N1	0.87 (5)	1.85 (5)	2.634 (3)	148 (4)

Symmetry codes: (i) −*x*+1/2, *y*−1/2, −*z*; (ii) −*x*+3/2, *y*+1/2, −*z*+1.
